# Secondary Metabolites Diversity of *Aspergillus unguis* and Their Bioactivities: A Potential Target to Be Explored

**DOI:** 10.3390/biom12121820

**Published:** 2022-12-06

**Authors:** Levy Tenório Sousa Domingos, Raquel dos Santos Martins, Leonardo Melo de Lima, Angela Michelato Ghizelini, Antonio Ferreira-Pereira, Fernando Cotinguiba

**Affiliations:** 1 Instituto de Microbiologia Paulo de Góes, Universidade Federal do Rio de Janeiro, Rio de Janeiro CEP 21941-902, Brazil; 2 Instituto de Pesquisas de Produtos Naturais Walter Mors, Universidade Federal do Rio de Janeiro, Avenida Carlos Chagas Filho, 373, Bloco H, Cidade Universitária, Rio de Janeiro CEP 21941-902, Brazil

**Keywords:** bioactive natural products, fungi metabolites, depsidones, depsides

## Abstract

*Aspergillus unguis* belongs to the Aspergillus section Nidulantes. This species is found in soils and organisms from marine environments, such as jellyfishes and sponges. The first chemical study reported in the literature dates from 1970, with depsidones nidulin (**1**), nornidulin (**2**), and unguinol (**3**) being the first isolated compounds. Fifty-two years since this first study, the isolation and characterization of ninety-seven (**97**) compounds have been reported. These compounds are from different classes, such as depsides, depsidones, phthalides, cyclopeptides, indanones, diarylethers, pyrones, benzoic acid derivatives, orcinol/orsenillate derivatives, and sesterpenoids. In terms of biological activities, the first studies on isolated compounds from *A. unguis* came only in the 1990s. Considering the tendency for antiparasitic and antibiotics to become ineffective against resistant microorganisms and larvae, *A. unguis* compounds have also been extensively investigated and some compounds are considered very promising. In addition to these larvicidal and antimicrobial activities, these compounds also show activity against cancer cell lines, animal growth promotion, antimalarial and antioxidant activities. Despite the diversity of these compounds and reported biological activities, *A. unguis* remains an interesting target for studies on metabolic induction to produce new compounds, the determination of new biological activities, medicinal chemistry, structural modification, biotechnological approaches, and molecular modeling, which have yet to be extensively explored.

## 1. Introduction

Many fungi are capable of producing heterogeneous low-molecular-mass compounds, also called secondary metabolites. These metabolites are not directly necessary for organism growth, unlike primary metabolites [1,2]. The versatile metabolism of fungi allows different types of interactions with other organisms, ranging from bacteria to metazoa, and substrates, which play essential roles in ecosystems. Over hundreds of millions of years, fungi use these metabolites as chemical signals for communication, defending their habitat, or inhibiting the growth of competitors, leading to the evolution of natural products and, consequently, acting in the ecological success of fungi in colonizing approximately all habitats on the planet [3,4,5,6].

Penicillin, considered the “wonder drug” of World War II and the first broad-spectrum antibiotic, was discovered in the fungus *Penicillium notatum* by Alexander Fleming in 1928. This metabolite significantly changed the search for new natural products from plants to microorganisms, inaugurating “the golden age of antibiotics [7,8,9]. Currently, 45 % of the known microbial metabolites are of fungal origin such as filamentous fungi, including *Penicillium*, *Trichoderma*, and *Aspergillus*, which represent almost 99 % of the total known fungal metabolites [10].

*Aspergillus* is an ascomycetous fungus with the greatest bioactive potential in nature. Species of this genus have been studied extensively for years, and they produce metabolites of considerable medical, industrial, agricultural, and economic importance. For example, the phytohormone gibberellin produced by *A. fumigatus* improves plant growth, and the anti-cholesterol drug lovastatin produced by *A. terreus*, with worldwide sales of USD 10 billion annually. The metabolic roles of these metabolites have been increasingly explored in biotechnological research that seeks new fungal natural products of commercial interest [11,12,13]. 

Fungal species such as *A*. *niger*, *A*. *oryzae*, and *A*. *terreus* are considered the work horses in biotechnology [14]. However, other species that are not in the spotlight and are less well known have great biotechnological value that has already been described, such as *A. unguis*. Research into this fungus include its application for the removal of heavy metals from industrial wastewater [15], expression systems for heterologous protein expression [16], production of enzymes of industrial interest [17,18] and, since the last century, the rich production of secondary metabolites. Therefore, this review highlights the secondary metabolites of *A*. *unguis* and their biological activities, with a brief description of the biology of *A*. *unguis*.

## 2. Biology of *A. unguis*

The fungus *A*. *unguis*, described in 1935 by Emile-Weill and L. Gaudin, belongs to the *Aspergillus* section *Nidulantes,* forming the series *Unguium* with four other species: *A. israelensis* [19], *A. croceus* [20], *A. longistipitatus* [21], and *A. croceiaffinis* [21], all of which were described between 2016 and 2020 using polyphasic taxonomy; that is, with the application of phenotypic, genotypic (using multigenic DNA sequences), and chemotaxonomic techniques [19,20,21,22]. *Aspergillus* taxonomy is complex, and it changes over time as species identification technology reorganizes just as new species are described [23]. In recent years, many efforts have been made by the scientific community to revise the taxonomy and phylogeny of several species that are already known through polyphasic taxonomy, mainly using DNA sequence data, in order to reorganize these thousands of species in light of new knowledge and modern techniques [24]. 

*Emericella unguis* is the sexual state (teleomorph) of *A. unguis* (anamorph), and it became synonymous with *A. unguis* with the new nomenclatural rules based on a single-name system established by the International Commission of *Penicillium* and *Aspergillus* (ICPA) in 2012 [24]. *A*. *unguis* has other possible synonyms, such as *Sterigmatocystis unguis* and *A. mellinus*, and there may be others; however, the differentiation of *A. unguis* and non-cleistothecial *A. nidulans* isolates was problematic in the past [20]. 

The complete *A. unguis* genome was sequenced in 2016 as part of a project to sequence *Aspergillus* species (Joint Genome Institute, https://jgi.doe.gov, accessed on 24 April 2022) [24]. This information may soon contribute to the taxonomic reordering of the species mainly by using the supporting information on metabolite production.

*A. unguis* can be isolated from soils [25], lichens [26], and organisms in marine environments, such as jellyfishes [27], seaweeds [28], sponges [29], and others. This ubiquitous fungus has great potential for the “one strain many compounds” (OSMAC) strategy, as it is capable of growing in culture broth with different sources of carbon and nitrogen, such as potato dextrose, oatmeal, glycerol casein, yeast extract sucrose, Czapek-Dox, and malt extract [30,31]. Solid media, in addition to solid agar media, are also used as the main media [26]. On some occasions, the medium can be supplemented with specific salts to study the assimilation and modulation of the production of secondary metabolites or with sea water to mimic the environment of marine isolates [29,30]. The use of *A*. *unguis* biology and its different growth methods are excellent alternatives for the discovery of new metabolites of this fungus.

## 3. Secondary Metabolism

**Figure 1:**
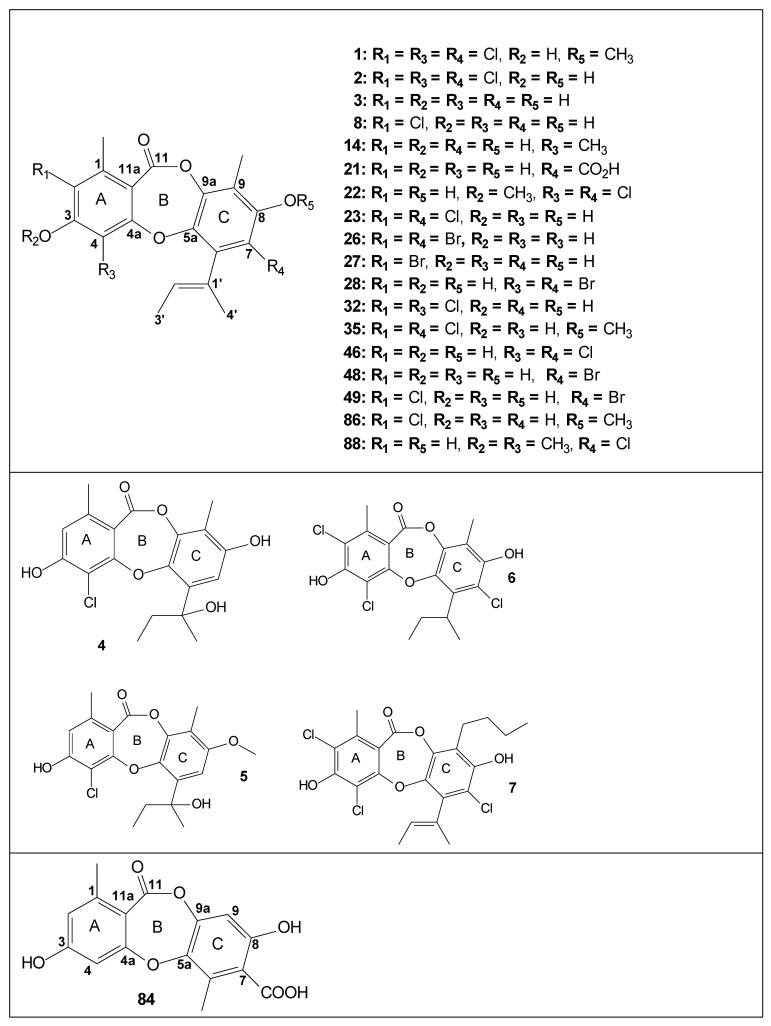

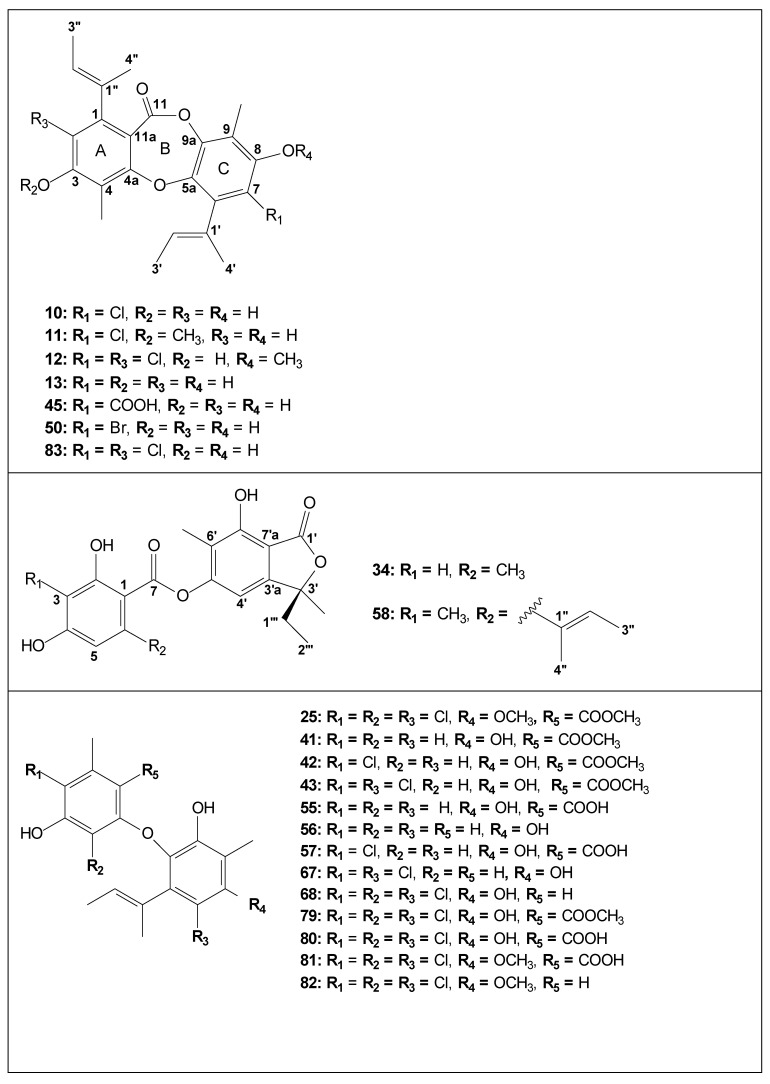

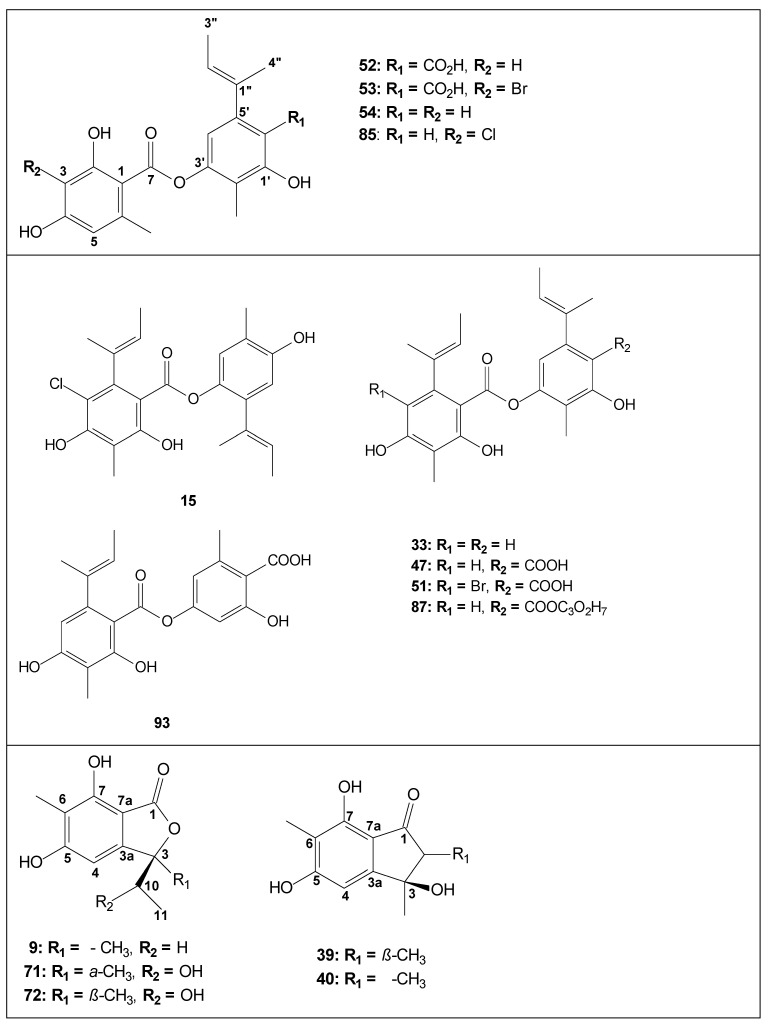

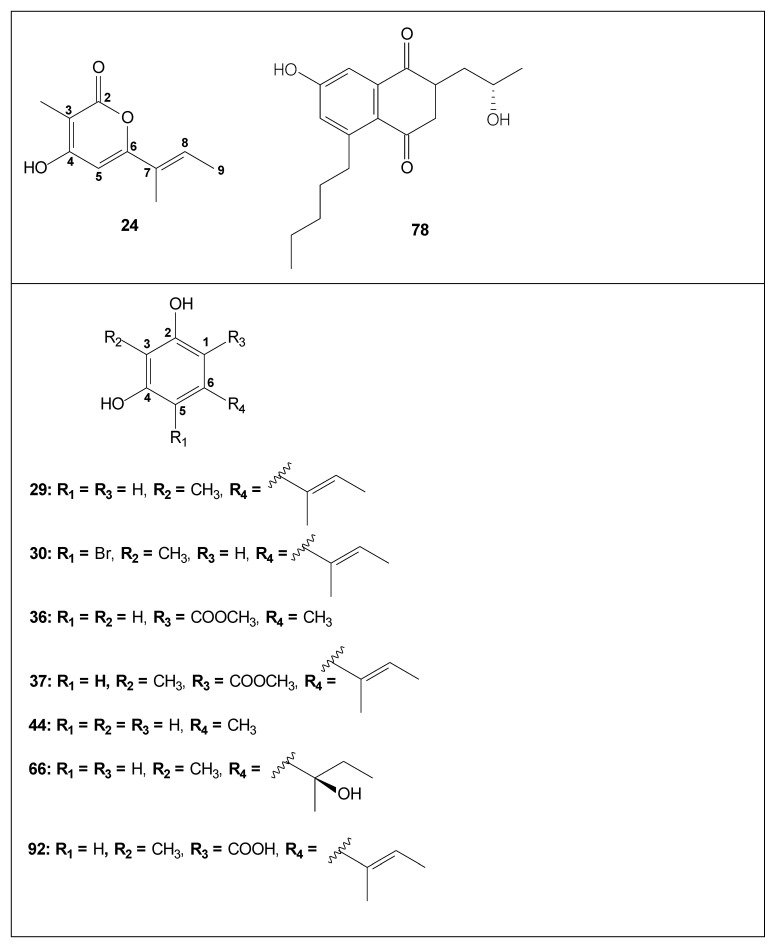

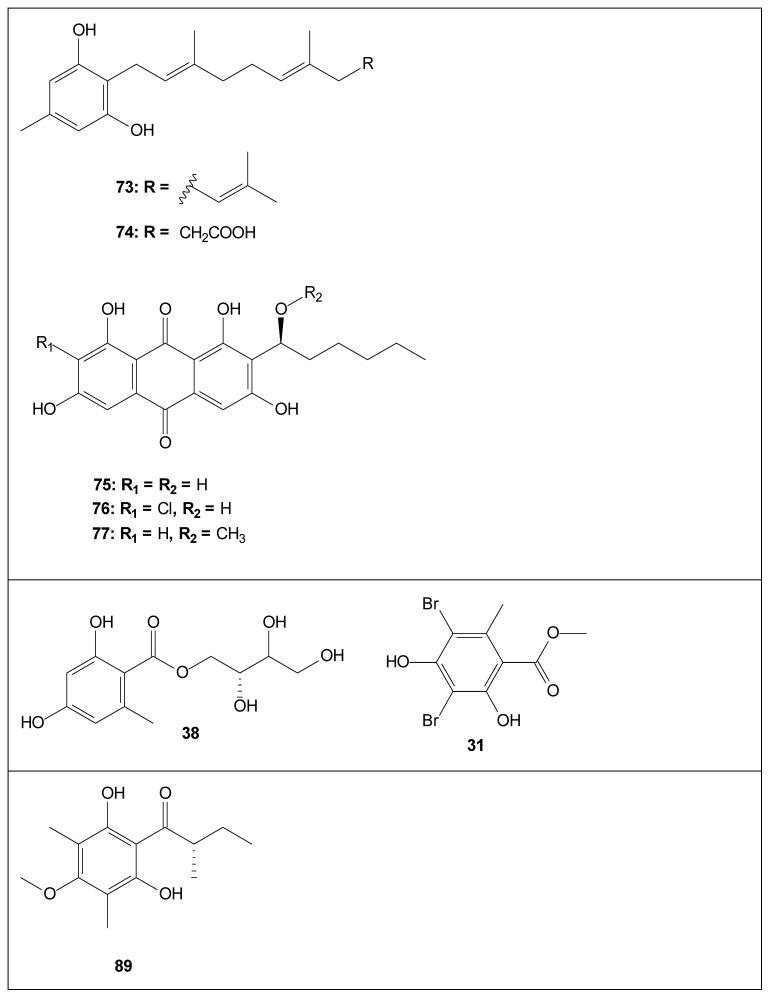

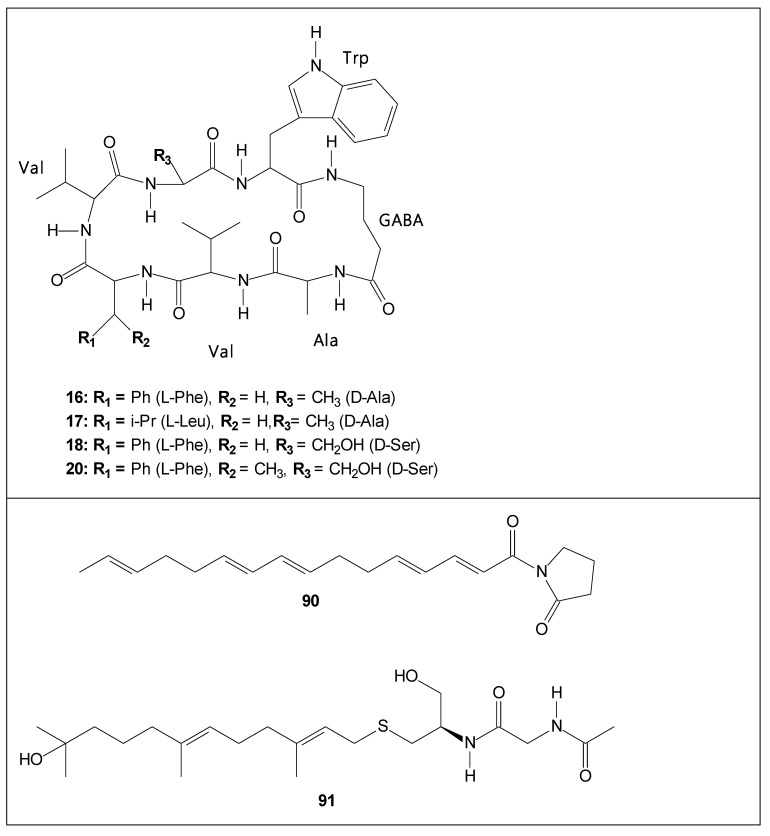

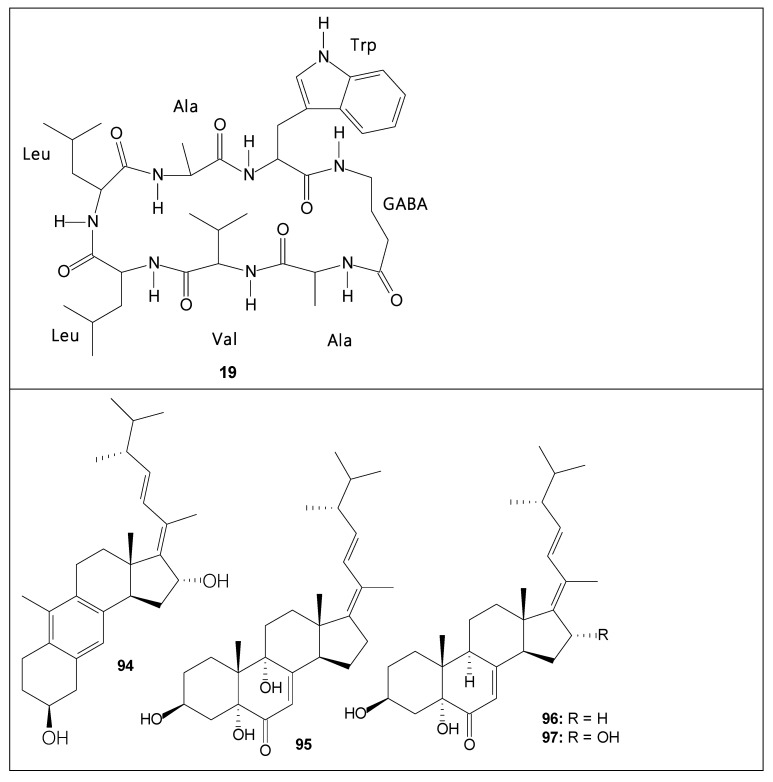

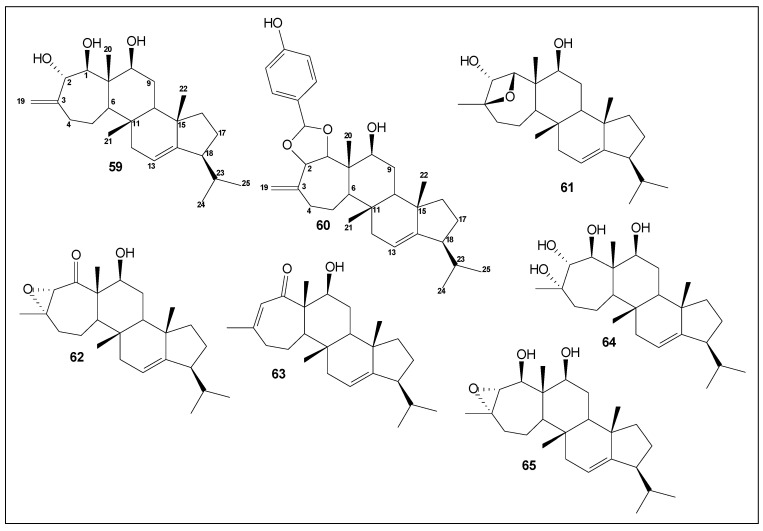
Depsidones (**1–8**, **10–14**, **21–23**, **26–28**, **32**, **35**, **39**, **45–46**, **48–50, 69–70, 83–84, 86, 88**), depsides (**15**, **33**, **47**, **51–54**, **85, 87, 93**), phthalides (**9**, **34**, **59, 71–72**), cyclopeptides (**16–20**), nitrogen-containing compounds (**90–91**), indanones (**40–41**), diarylethers (**25**, **41–43**, **55–57, 67–68, 79–82**), pyrones (**24**), benzoic acid derivative (**31**), orcinol/orsenillate derivatives (**29–30**, **36–38**, **44, 66, 73–74, 89, 92**), anthraquinones (**75–77**), chromone (**78**), sesterpenoids (**59–65**) and sterols (**94–97**) from *A. unguis*.

A review of the 52-year-long chemistry studies of *A. unguis* (1970–2022) was performed, from the first articles published by Kamal in 1970 to the last published by Cao in 2022 [32,33]. In this review, ninety-seven chemical structures of compounds isolated and identified from *A. unguis* are reported. In Table 1, these compounds are organized by code, presenting the following information: (I) the class of metabolites to which they belong, (II) the biological activities of each compound, and (III) the references that report the isolation and identification. The compounds found in *A. unguis* are classified into depsides, depsidones, phthalides, cyclopeptides, indanones, diarylethers, pyrones, benzoic acid derivatives, orcinol/orsenillate derivatives, and sesterpenoids. Most of the compounds are depsidones, representing approximately 30 % of the total. Among the ninety-seven compounds isolated from *A unguis*, only twelve were also isolated from other microorganisms: nidulin (**1**), nornidulin (**2**), unguinol (**3**), 2-chlorounguinol (**8**), (3*S*)-3-ethyl-5,7-dihydroxy-3,6-dimethylphthalide (**9**), folipastatin (**13**), unguisin E (**20**) aspergillusidone C (**23**), pilobolusate (**37**), (+)-montagnetol (**38**), averantin (**75**), and corynesidone D (**84**) (see Table 1). The chemical structure of each compound is also presented in Figure 1.

A549 = human lung carcinoma, AChE = acetylcholinesterase, BS = *Bacillus subtilis* (ATCC 6633 or KCTC 1021), CA = *Candida albicans* NCPF3153, CN = *Cryptococcus neoformans* ATCC90113, HepG2 = human hepatocellular liver carcinoma, HT-29 = human colorectal adenocarcinoma cell line, HuCCA-1 = human cholangiocarcinoma, KB = human oral carcinoma cell line, MRSA = methicillin-resistant *Staphylococcus aureus*, MCF-7 = breast cancer cells, MG = *Microsporum gypseum* clinical isolate, MOLT-3 = acute lymphoblastic leukemia, NS-1= mouse myeloma cells (ATCC TIB-18), PA = *Pseudomonas aeruginosa*, PN = *Penicillium marnefeii*, SA = *Staphylococcus aureus* (ATCC25923, or ATCC29213, or KCTC 1927), SC = *Saccharomyces cerevisiae* (ATCC 9763), SMMC-7721 = human hepatoma cells, SOAT1 = sterol O-acyltransferase ubiquitously expressed in all tissues and cells, SOAT2 = sterol O-acyltransferase expressed predominantly in the liver (hepatocytes) and intestine, U251 = glioblastoma cell line, U87 = human primary glioblastoma cell, PF = *Plasmodium falciparum* (K1, multidrug-resistant strain), MT = *Mycobacterium tuberculosis* (ATCC25177); BC = *Bacillus cereus* (ATCC11778); AB = *Alternaria brassicicola* (BCC42724); CA = *Colletotrichum acutatum* (BCC58146); ML = *Micrococcus luteus* (KCTC 1915); MV = *Microbulbifer variabilis*; MJ = *Marinobacterium jannaschii*; VP = *Vibrio pelagius*.

### 3.1. Depsidones

Depsidones consist of two aromatic rings (A and C rings) joined by a -CO-O- bridge (ester group) and an ether group, forming a third seven-membered ring (B ring). This class of compounds is biosynthesized through oxidative coupling of the depsides [42,70]. 

Three depsidones, nidulin (**1**), nornidulin (**2**), and yasimin (**3**), were isolated [33]. In the same year, the authors published a biosynthetic study of yasimin (**3**) by incorporating labeled acetate (1-^14^C and 2-^14^C) and malonate-1-^14^C using a fungus culture growing in Czapek-Dox medium [54]. The labeled compound **3** was isolated, and the authors were able to make a series of considerations about the biosynthesis pathways of this compound, classifying them as polyketides [41,54]. Furthermore, in 1970, Kamal published another study describing the identification of four new depsidones from *A. unguis*: haiderin (**4**), rubinin (**5**), (-)-shirin (**6**), and nasrin (**7**). None of the four compounds described in this study were reisolated from *A. unguis* or any other species of fungus; therefore, this is the only report of these four compounds in literature. 

Stodola et al. reisolated compound **3** in 1972 but did not publish the results; however, it was renamed unguinol (**3**) [56]. Since then, most articles have referred to this compound as unguinol, not yasimin, despite having exactly the same chemical structure. In 1983, Turner and Aldridge identified nidulin (**1**), nornidulin (**2**), and unguinol (**3**) in *E. nidulans* (anamorph *A. nidulans*) and *A. mellinus*; however, these two organisms have been reidentified as *A. unguis* [27]. In 1988, Kawahara et al. published two studies describing a series of new depsidones, including emeguisin A (**10**), emeguisin B (**11**), and emeguisin C (**12**) [38,39]. These compounds are the first examples of depsidones bearing two 1-methylprop-1-enyl groups in one molecule. Uchida et al. described the structure of a supposedly unprecedented compound, which they named 7-chlorofolipastatin (**10**). However, this compound was previously identified as emeguisin A (**10**) [47]. Sureram et al. (2013) subjected *A. unguis* to growth in medium containing different halogen salts (potassium bromide [KBr], potassium iodide [KI], and potassium fluoride). The fungus grown in medium containing KBr produced three new brominated depsidones, namely aspergillusidones D–F (**26–28**). Meanwhile, when the fungus was subjected to medium containing KI, the fungus did not incorporate iodine atoms into the compounds, but a new depsidone, 2,4-dichlorounguinol (**32**) was isolated and identified. Morshed et al. (2018) manipulated the concentration of halides in the culture medium, inducing *A. unguis* to produce a series of new compounds, by OSMAC strategy. When the fungus was grown in yeast extract with supplements (YES) medium without saline supplementation, several compounds were produced in addition to an unprecedented substance, 7-carboxyfolipastatin (**45**). When the YES medium was supplemented with 0.5 % sodium chloride, it was possible to decrease the production of unguinol (**3**), in addition to producing new compounds such as **45** and 4,7-dichlorounguinol (**46**). In an experiment with 0.5 % KBr supplementation, the fungus produced new substances, such as 7-bromounguinol (**48**), 2-chloro-7-bromounguinol (**49**), and 7-bromofolipastatin (**50**). Sureram (2013) and Morshed (2018) explored the concept of precursor-directed biosynthesis, which is an attempt to exploit the metabolic potential of fungi in the face of a precursor. Modifying the concentration of the halides is a powerful tool for modulating secondary metabolite production and triggering quiescent pathways in the fungus. The fungus must be able to recognize, metabolize and generate new unnatural structures [71]. Sureram (2013) and Morshed (2018) showed that this approach was efficient in generating new structures of secondary metabolites. The structural diversity of depsidones has increased due to the recent discoveries of Saetang et al. (2021). They described two new depsidones (**69** and **70**), both harboring an interesting structure different from all other depsidones: the substitution of the 2-butenyl unit (C ring) for the hydroxy-3-butenyl moiety group. These recent discoveries show that the metabolic potential of *A. unguis* remains to be elucidated and may elicit surprise.

### 3.2. Depsides

Depsides are substances that are biosynthesized from the union of two orsellinic acid derivative units or the union of an orsellinic acid derivative and an orcinol derivative [42]. Depsides are related to depsidones and are generally accepted as biosynthetic precursors of this depsidones [43]. Nielsen et al. (1999) isolated and identified the first depside in *A. unguis*: guisinol (**15**). After almost 20 years, Phainuphong et al. (2018) described the identification of three new depsides: aspergiside A (**54**), aspergiside B (**52**), and aspergiside C (**58**). Coincidentally, Morshed et al. (2018) also described the isolation and structural characterization of compounds **52** and **54**, but they called these structures unguidepside A (**52**) and decarboxyunguidepside A (**54**). Yang et al. (2018) used a plasma-induced mutant and the combined use of an epigenetic modifier (procaine) with sodium bromide as a strategy to diversify the production of secondary metabolites by a marine strain of *A. unguis*. Ten compounds were isolated and identified, one of which was a new depside named aspergillusidone G (**52**). However, this compound is structurally identical to those described by Phainuphong et al. (2018) and Morshed et al. (2018): aspergiside B (**52**) and unguidepside A (**52**), respectively. Yang et al. (2018) classified aspergillusidone G (**52**) as a depsidone, but the other authors classified it as a depside [25,28,30].

### 3.3. Phthalides

Phthalide or isobenzofuran-1(3H)-one is a chemical group that joins an aromatic ring and a dihydrofuranone group. The 3-ethyl-5,7-dihydroxy-3,6-dimethylphthalide (**9**) was the first phthalide isolated from *A. unguis*. This compound is also considered the first example of a 3,3-disubstituted phthalide obtained from a natural source [38]. However, there are still a few descriptions of this class of compounds in *A. unguis*, especially the following: asperlide (**34**), aspergiside C (**58**), asperunguislide A (**71**), and asperunguislide B (**72**). 

### 3.4. Cyclopeptides

Cyclic heptapeptides unguisin A (**16**) and unguisin B (**17**) isolated from a marine-derived strain of *E. unguis* are considered to contain gamma-aminobutyric acid (GABA) in the ring. The only difference between the two peptides is that unguisin A (**16**) contains L-phenylalanine, while unguisin B (**17**) has L-leucine [55]. The cyclopeptide unguisin C (**18**) was also isolated as a minor component in 2002 [63]. This peptide is structurally similar to unguisin A (**16**), except for the substitution of D-alanine to D-serine, yielding a more hydrophilic peptide. Unguisin D (**19**) was produced when L-leucine was added to the fermentation medium and was detected only by liquid chromatography mass spectrophotometry analysis (LC-MS), with the structure cyclo-(tryptophyl-GABA-alanyl-valyl-leucyl-leucyl-alanyl). This compound is similar to unguisin B (**17**) with the replacement of valine-2 to leucine [63]. In 2011, Liu and Chen (2011) reported the isolation of a new cyclopeptide, unguisin E (**20**), from *Aspergillus* sp. AF119 [64]. The only difference was that the amino acid phenylalanine in unguisin A (**16**) was replaced by β-methylphenylalanine in **20**.

### 3.5. Pyrone

Pyrones are a class of heterocyclic chemical compounds that contain an unsaturated six-membered ring containing one cyclic ester functional group. Despite its presence in fungi, the metabolism of pyrones is still not well known in *A. unguis;* only one compound of this class has been isolated and identified. The 3-methyl-4-hydroxy-6-(1-trans-methyl-1-propenyl)-2-pyrone (**24**) was isolated as a natural source for the first time. This pyrone was a des-O-methyl derivative of nectriapyrone, a metabolite previously isolated from the fungus *Gyrostroma missouriense* [29].

### 3.6. Diarylethers

Several diarylethers were isolated from *A. unguis* from 2010. The first was an unprecedented compound described by Sureram et al. (2012) as aspergillusether A (**25**). The authors considered the possibility that compound **25** could be an artifact obtained from the cleavage of an ester bond of nidulin (**1**), followed by methylation at the carboxylic group, which possibly occurred during extraction and purification. Subsequently, several new diaryethylethers were isolated as aspergillusethers B-D (**41–43**) [31], unguolic acid (**55**), decarboxyunguolic acid (**56**), 5-chlorounguinolic acid (**57**) [30] and aspergillusethers E–F (**67–68**) [41]. 

### 3.7. Orcinol/Orsenillate Derivatives and Benzoic Acid Derivatives

Structurally, orcinol consists of an aromatic ring interspersed by two hydroxyls and one methyl group. Orcinol (**44**), despite being a known substance, has not yet been reported in *A. unguis* [31]. Two new orcinol derivatives, aspergillusphenol A (**29**) and aspergillusphenol B (**30**), were identified by Sureram et al. (2013). Orselinates had not been reported in *A. unguis* until the study by Klaiklay et al. (2017) who identified methyl orsellinate (**36**), pilobolusate (**37**), and (+)-montagnetol (**38**). The (+)-montagnetol is commonly isolated from lichen, especially from the genus *Roccella* [72].

### 3.8. Indanones

Phainuphong described the isolation and structural characterization of a class of compounds that had not yet been identified in *A. unguis*: indanones. Two new indanones, asperunguisone A (**39**) and asperunguisone B (**40**), were described. Indanones structurally exhibit a junction between an aromatic ring and a cyclopropane ring with a ketone function [31]. 

### 3.9. Anthraquinones

Anthraquinones (polyketides compounds) are an important chemical group. The skeleton is basically two aromatic rings separated by two carbonyl groups. Although these compounds have already been isolated from other species of the genus *Aspergillus*, it was only in 2021 that the anthraquinones averantin (**75**), 7-chloroaverantin (**76**) and 1’-O-methylaverantion (**77**) were isolated from strains 158SC-067 of *A. unguis* [68].

### 3.10. Terpenoids and Sterols

Terpenoids belong to a class of compounds formed by the coupling of isoprene units (dimethylallyl pyrophosphate and isopentenyl pyrophosphate) and are classified into subclasses according to the multiplicity of five carbons. In fungi, these isoprene units originate from the mevalonate pathway. Li et al. (2019) isolated and identified six new asperane-type sesterterpenoids (terpenes with 25 carbon atoms), asperunguisins A–F (**59–64**), together with a known analogue, aspergilloxide (**65**). These are rare asperane-type of sesterterpenoids, characterized by a unique hydroxylated 7/6/6/5 tetracyclic system. Compound **65** was discovered as the first asperane-type sesterterpenoid from the marine-derived fungus *Aspergillus* sp. in 2002 [73]. Rare ergostane-type sterols with an unusual unsaturated side chain (**94–97**) were reported by Cao and collaborators [32]. It was the first time that this type of compound had been isolated from *A. unguis*.

## 4. Biological Potential of Natural Products Isolated from *A. unguis*

Several biological activities were attributed to the various compounds isolated from *A. unguis*: enzymatic inhibition (acetylcholinesterase, aromatase, phospholipase A, sterol O-acyltransferase, xanthine oxidase), activity against cancer cell lines (A549, HB, HuCCA-1, HepG2, HT-29, KB, MCC-7, MOLT-3, NS-1, SMMC-7721, T47D, U251, U87, Vero), antibacterial properties (against *Bacillus subtilis*, *Staphylococcus aureus*, methicillin-resistant *S. aureus*, *Pseudomonas aeruginosa*), antifungal properties (against *Candida albicans*, *Cryptococcus neoformans*, *Microsporum gypseum*), herbicidal effects, larvicidal effects (against brine shrimp larva lethality test), DNA repair activity, animal growth promotion, antimalarial properties, and antioxidant activities. Table 1 presents data on the biological activities of all ninety-seven compounds already identified in *A. unguis*. All this information shows the immense potential, indicating the ways that can be better explored and others that have not been investigated.

### 4.1. Antifungal, Antibacterial, Antimalarial and Larvicidal Activities

Considering the tendency for antibiotics to become ineffective against resistant strains, *A. unguis* compounds have also been extensively investigated and some of them are very promising against larvae and pathogenic microorganisms. A good example is the study by Yang et al. (2022) which show the potent activity of depsidones nornidulin (**2**), emeguisin A (**10**) and folipastatin (**13**) against vancomycin-resistant bacteria *Enterococcus faecium* [44]. Synthetic derivatives of nidulin (**1**) were also investigated for their significant activities against Gram-positive bacteria, including methicillin-resistant *Staphylococcus aureus* bacterial properties. These derivatives, in particular 8-O-aryl ether derivatives, proved to be very promising [74]. 

Saetang et al. pointed out the activity of the depsides **10** and **13** that were strongly active against the pathogenic yeast *Candida neoformans* with respective MIC values of 1 and 0.5 μg/mL, an identical value to the positive control, the antifungal amphotericin B. Another despside, aspergillusidone C (**23)** showed the strongest antifungal activity against *Microsporum gypseum* with the MIC value of 2 μg/mL, similar to positive control clotrimazole. 

Yang et al. (2018) considers that the compounds nidulin (**1**), nornidulin (**2**) and aspergillusidone F (**28**) could be used for the development of pesticides for their larvicidal activity. These depsidones exhibited potent larvicidal activity against brine shrimp, with close or even lower LC_50_ values compared with the positive control mercury (II) nitrate, Hg(NO_3_)_2_ [31]. Klaiklay and colleagues also demonstrated the potent antimicrobial activities of the depsidone emeguisin A (**13**). This compound **13** exhibited the most potent antibacterial (against *S. aureus* and methicillin-resistant *S. aureus*), and antifungal (against *C. neoformans*) activities with MIC values of 0.5 µg/mL. Emeguisin A (**13**) also showed antimalarial activity, with MIC values of 2.2 µM against *P. falciparum,* identical to the positive control dihydroartemisinin [40].

### 4.2. Anti-Osteoclastogenic Activity

With the aging of the population, some diseases such as osteoporosis have become a concern in recent times. Therefore, the search for new bioactive molecules that can be applied to treatments and therapies becomes extremely important and some research groups have moved towards this. Zhang et al. (2022) evaluated several pure substances and guisinol (**15**) proved to be a further dose-dependent suppressed RANKL-induced osteoclast differentiation without any evidence of cytotoxicity in bone marrow macrophage cells. This is the first article that reports the identification of an *A. unguis* compound that has this type of activity [46].

### 4.3. Cytotoxic Activity against Cancer Cell Lines

Cancer is one of the most feared diseases and its treatment can still leave the patient debilitated and sometimes discouraged. Compounds isolated from *A. unguis* have been extensively evaluated against several tumor cell lines. As an example, Cao et al. (2022) evaluated ergosterane-type sterol against ACHN (renal), HCT-15 (colon), NUGC-3 (stomach), PC-3 (prostate), NCI-H23 (lung), and MDA-MB-231 (breast) cancer cell lines. The new substance aspersterol A (**94**) showed cytotoxicity against all the tested cell lines [32]. The orsellinate pilobolusate (**37**) exhibited potent activity against the KB cell line with an IC_50_ value of 4.5 µM which was much stronger than the standard drug, ellipticin [40]. The asperane sesterterpenoid asperunguisin C (**61**) showed cytotoxicity against the human cancer cell line A549 with an IC50 value of 6.2 μM, a value very close to the positive control adriamycin (2.9 μM) [26].

### 4.4. Anti-Inflammatory Activity

Cao and colleagues monitored the anti-inflammatory potential of ergostanes and concluded that the compound aspersterol C (**96**) showed the most potent anti-inflammatory activity. This compound inhibited the production of inflammatory mediators, including IL-6 and iNOS in LPS-induced macrophages (RAW 264.7 cells). The authors consider that this molecule could be used as a lead for additional studies for anti-inflammatory models [32]. In a similar study, this research group isolated *A. unguis* and the anti-inflammatory properties of compound variotin B (**90**), the first linear nitrogenous secondary metabolite isolated from *A. unguis* [62].

### 4.5. Enzyme Inhibitors

Biological tests involving natural products were greatly advanced in the 1990s. Among all the studies analyzed, the first one that shows the attribution of biological activity to a compound isolated directly from *A. unguis* was reported by Hamano and collaborators in 1992, wherein depsidone folipastatin (**13**) inhibited the enzyme phospholipase A_2_ enzyme isolated from rabbit peritoneal exudate. These enzymes are involved in inflammatory processes [52]. Studies suggest that unguinol inhibits C4 plant enzyme pyruvate phosphate dikinase (PPDK) via a novel mechanism of action which also translates to a herbicidal effect on whole plants. This compound had deleterious effects on a model C4 plant but no effect on a model C3 plant [49].

The depsidone aspergillusidone C (**23**) showed the inhibitory activity of the aromatase enzyme with an IC_50_ value of 0.74 µM, being more potent than the positive standard ketoconazole (IC_50_ value 2.4 µM). It was concluded, from the structure activity relationship view, that the depsidone structure is important for this type of activity. The aromatase enzyme participates in aromatization reactions from androgens to the aromatic ring of estrogens and the inhibition of this enzyme reduces the incidence of breast cancer [29]. The same research group showed in another publication that unguinol (**3**) and aspergillusidone A (**21**) are also inhibitors of this enzyme [50]. The depsidones unguinol (**3**) and aspergillusidone D (**26**) were selected for further studies. Unguinol (**3**) induced apoptosis and cell cycle arrest in the breast cancer cell line-MB 231. Unguinol (**3**) and aspergillusidone D (**26**) also inhibit lifetime cell viability at low concentrations (μM) [51].

Depsidones were also evaluated for their potential to inhibit the enzyme acetylcholinesterase (AChE). Aspergillusidone A (21) showed AChE inhibition with IC50 value of 56.8 μM, with donepezil used as a positive control (IC50 = 0.3 μM). Docking studies were also performed, showing that this depsidone interacts with the enzyme in different ways [28].

## 5. Summary and Future Perspectives

In the past fifty-two years (1970–2022), since the first chemical study of *A. unguis*, ninety-seven compounds from different classes of microbial secondary metabolites have been isolated and identified by spectrometric and spectroscopic techniques. Many of the reported compounds are produced exclusively by *A. unguis*. The substances isolated from *A. unguis* have promising biological activity against pathogens such as larvae, bacteria and yeasts, with several examples where these substances are even more active in in vitro assays than the positive controls. Considering the tendencies of antiparasitic and antibiotics to become less effective, it is very important to continue these studies in order to investigate their action in vivo. In addition to antimicrobial and larvicidal activities, these substances also demonstrate promising activities against the cancer cell lineage, anti-inflammatory, and antimalarial activities. Several compounds of *A. unguis* have also been shown to be enzyme inhibitors, such as aromatase and AChE. The metabolic potential of *A. unguis* is still largely untapped; therefore, it has great potential for innovation. Variations in the concentration or depletion of salts in the culture media proved to be an extremely interesting approach to produce new structures. The OSMAC strategy, despite having been successfully applied by several authors in *A. unguis*, can still be widely explored, changing the parameters of microorganism cultures, in order to allow the fungus to produce new chemical structures [28,30,68]. Approaches using epigenetic regulation also have not been widely used in *A. unguis*. Epigenetic regulation is critical for fungal secondary metabolism biosynthesis and the activation of gene clusters.The modulation of epigenetic regulation is an interesting alternative for discovering new secondary metabolites and improving their production [75]. Another tool that could also accelerate the discovery of new minority metabolites as well as understand the metabolic capacity of *A. unguis* are metabolomics-based dereplication methods. These approaches have not yet been applied to this fungus and deserve attention [76]. Medicinal chemists and molecular modeling specialists could also contribute significantly to the understanding of the interaction of secondary metabolite X enzyme inhibition. Despite the diversity of chemical structures, few studies have focused on the structural modification of these compounds which deserve attention in order to obtain semisynthetic derivatives even more potent than the natural substances.

## Figures and Tables

**Table 1:** All the compounds identified in *A. unguis* were organized by code, showing for each one (I) the references that report the isolation and identification; (II) the chemical class to which they belong and (III) the biological activities of each compound.

Code	Compound	Biological Activities	Isolation and Identification
**Depsidones**			
**1**	nidulin	Inhibition of xanthine oxidase [29]; aromatase inhibitory activity [29]; HuCCA-1, HepG2, A549, MOLT-3 cancer cell line (weak cytotoxicity) [29]; anti-MRSA and brine shrimp larva lethality test strong bioactivity [34]; DNA-damage repair test (anti-AB3027) [34]; antimicrobial activity against BS and SA [30]; antimicrobial activity against SA and MRSA [25]; larvicidal activity using brine shrimp model [28]; antimicrobial activity against BC [35]; anti-phytopathogenic activity against CA [35]; growth inhibition against human cancer cell lines [36]	[25,28,29,30,33,34,35,36,37,38,39,40,41,42,43,44] also isolated from *A. nidulans* [45]
**2**	nornidulin	Inhibition of xanthine oxidase [29]; aromatase inhibitory activity [29]; HuCCA-1, HepG2, A549, MOLT-3 cancer cell line (weak cytotoxicity) [29]; anti-MRSA and brine shrimp larva lethality test [34]; DNA-damage repair test (anti-AB3027) [34]; antimicrobial in SA, MRSA, and CN [42]; antimicrobial activity against BS and SA [30]; antimicrobial activity against SA, MRSA and CN [25]; larvicidal activity/brine shrimp model [28]; antimicrobial activity against BC [35]. anti-phytopathogenic activity against AB and CA [35]; growth inhibition against human cancer cell lines [36]; inhibition effect on LPS-induced NF-kβ activation [46]. Antimicrobial activity against MRSA, MV, MJ and VP [46]; antimicrobial activity against Gram-positive and Gram-negative bacteria [41]	[25,27,28,29,30,33,34,35,36,40,41,42,43,44,46,47] also isolated from *A. nidulans* [45]
**3**	unguinol (yasimin)	Animal growth permittant [48]; pyruvate phosphate dikinase (PPDK) inhibitor (a potential herbicide target) [49]; aromatase inhibitory activity [37]; HuCCA-1, HepG2, A549, MOLT-3 cancer cell line (weak cytotoxicity) [37]; KB, MCF-7 and Vero cancer cell line (cytotoxicity) [42]; T47D tumor cells most likely via inhibition of aromatase (CYP19) activity [50]; SOAT1 and SOAT2 isozymes inhibition [47]; antimicrobial activity against BS [30]; apoptosis induction and cell cycle arrest in MDA-MB-231 cells induction [51]; antimicrobial activity against MT [35]; anti-phytopathogenic activity against AB [35]; growth inhibition against human cancer cell lines [36]; inhibition effect on LPS-induced NF-kβ activation [46]	[25,28,30,33,35,36,38,39,40,41,42,43,46,47,48,49,50,52,53,54,55,56] also isolated from *Aspergillus nidulans* and *Trichoderma asperellum* [57,58]
**4**	haiderin	-	[53]
**5**	rubinin	-	[53]
**6**	(-)-shirin	-	[53]
**7**	nasrin	-	[53]
**8**	2-chlorounguinol	Inhibition of xanthine oxidase [29]; aromatase inhibitory activity [29]; antimicrobial in SA, MRSA, CA, CN and MG [42]; SOAT1 and SOAT2 isozymes inhibition [47]; antimicrobial activity against SA, MRSA, CA, and MG [25]; antimicrobial activity against PA and MRSA [28]; larvicidal activity/brine shrimp model [28]; antimicrobial activity against MT, BC [35]. Anti-phytopathogenic activity against AB and CA [35]; growth inhibition against human cancer cell lines [36]	[25,28,29,30,35,36,38,39,40,41,42,44,47,48] also isolated from *Trichoderma asperellum* [58]
**10**	emeguisin A/7-chlorofolipastatin	Antimicrobial in SA, MR*SA* and CN [42]; antimalarial activity against PF [35,42]; antimicrobial activity against BS, SA and SC [30]; antimicrobial activity against SA, MRSA, CA and CN [25]; cytotoxic activity against KB and Vero cells [25]; SOAT1 and SOAT2 isozymes inhibition [47]; antimicrobial activity against MT, BC [35]; anti-phytopathogenic activity against CA [35]; growth inhibition against human cancer cell lines [36]; antimicrobial activity against Gram-positive and Gram-negative bacteria [41]	[25,30,35,36,39,40,41,44]
**11**	emeguisin B	Antimalarial activity against PF [35]	[35,39,41,44]
**12**	emeguisin C	Antimalarial activity against PF [35]; Antimicrobial activity against MT, BC [35]	[35,39]
**13**	folipastatin	Phospholipase A inhibitor [55]; antimicrobial in SA, MRSA, and CN [42]; antimalarial activity against PF [35,42]; SOAT1 and SOAT2 isozymes inhibition [47]; antimicrobial activity against BS and SA [30]; cytotoxity against NS-1 [30]; antimicrobial activity against SA, MRSA and CN [25]; cytotoxic activity against Vero cells [25]; growth inhibition against human cancer cell lines [36]; antimicrobial activity against Gram-positive and Gram-negative bacteria [41]	[25,30,35,36,40,41,44,47,48,52] also isolated from *Wicklowia aquatica* [59]
**14**	4-methylunguinol	-	[48]
**21**	aspergillusidone A	Inhibition of superoxide anion radical formation by xanthine/xanthine oxidase [29]; aromatase inhibitory activity [29]; HuCCA-1, HepG2, A549, MOLT-3 cancer cell line (only weak cytotoxicity) [29]; T47D tumor cells most likely via inhibition of aromatase (CYP19) activity [50]; antimicrobial activity against MRSA and CA [28]. AChE inhibitory activity [28]. Larvicidal activity using brine shrimp model [28]; anti-phytopathogenic activity against CA [35]	[25,28,29,35,41,44,50]
**22**	aspergillusidone B	Inhibition of superoxide anion radical formation by xanthine/xanthine oxidase [29]; aromatase inhibitory activity [29]; antimalarial activity against PF [35]; antimicrobial activity against BC [35]; inhibition effect on LPS-induced NF-kβ activation [46]	[25,29,35,44,46]
**23**	aspergillusidone C(2,7-dichlorounginol)	Aromatase inhibitory activity [29]; inhibition of xanthine oxidase [29]; HuCCA-1, HepG2, A549, MOLT-3 cancer cell line (only weak cytotoxicity) [29]; anti-A549 tumor cell line [34]; anti-MRSA and brine shrimp larva lethality test strong bioactivity [34]; DNA-damage repair test (anti-AB3027) [34]; antimicrobial in SA, MRSA, CA, CN and MG [42]; antimalarial [42]; VERO cell line (strong cytotoxicity) [42]; antimicrobial activity against BS, SA and SC [30]; antimicrobial activity against SA, MRSA, and MG [25]. Cytotoxic activity against Vero cells [25]; antimicrobial activity against PA [28]. Larvicidal activity using brine shrimp model [28]; antimicrobial activity against MT, BC [35]; anti-phytopathogenic activity against AB and CA [35]; growth inhibition against human cancer cell lines [36]; antimicrobial activity against MRSA, MV, MJ and VP [46];	[25,28,29,30,34,35,36,40,41,44,46,47] also isolated from *Trichoderma asperellum* [58]
**26**	aspergillusidone D	Aromatase inhibitory activity [37]; HuCCA-1, HepG2, A549, MOLT-3 cancer cell line (only weak cytotoxicity) [37]; antimicrobial activity against BS, SA and SC [30]; apoptosis induction and cell cycle arrest in MDA-MB-231 cells induction [51]	[30,42]
**27**	aspergillusidone E	Aromatase inhibitory activity [37]; HuCCA-1, HepG2, A549, MOLT-3 cancer cell line (only weak cytotoxicity) [37]; antimicrobial activity against BS, SA and SC [30]	[30,42]
**28**	aspergillusidone F	Aromatase inhibitory activity [37]; HuCCA-1, HepG2, A549, MOLT-3 cancer cell line (only weak cytotoxicity) [37]; antimicrobial activity against BS, SA and SC [30]; antimicrobial activity against PA and MRSA [28]. Larvicidal activity using brine shrimp model [28].	[28,30,42]
**32**	2,4-dichlorounguinol	Antimicrobial activity against SA and MRSA [25]	[25,42]
**35**	aspersidone	Antimicrobial activity against SA and MRSA [25]. Cytotoxic activity against Vero cells [25]; antimicrobial activity against BC [35]; anti-phytopathogenic activity against AB and CA [35]; growth inhibition against human cancer cell lines [36]	[25,35,36,40,41]
**45**	7-carboxifolipastatin	-	[30,44]
**46**	4,7-dichlorounguinol	Antimicrobial activity against BS, SA and SC [30]	[30]
**48**	7-bromounguinol	Antimicrobial activity against BS, SA and SC [30]	[30]
**49**	2-chloro-7-bromounguinol	Antimicrobial activity against BS, SA and SC [30]	[30]
**50**	7-bromofolipastatin	Antimicrobial activity against BS and SA [30]	[30]
**69**	asperunguissidone A	Antimicrobial activity against SA and MRSA [38]	[41]
**70**	asperunguissidone B	-	[41]
**83**	emeguisin D	Antimalarial activity against PF [35]; antimicrobial activity against MT, BC and SA [35];	[35]
**84**	corynesidone D	Antimicrobial activity against BC [35];	[35] also isolated from *Corynespora cassiicola* [60]
**86**	aspersidone B	Antimicrobial activity against BS, ML and SA [36]; growth inhibition against human cancer cell lines [36]	[36]
**88**	aspergillusidone H	Inhibition effect on LPS-induced NF-kβ activation [46]	[46]
**Depsides**			
**15**	guisinol	Growth inhibition against human cancer cell lines [36]; antimicrobial activity against MRSA, MV, MJ and VP [46]	[27,36,46]
**33**	agonodepside A	Growth inhibition against human cancer cell lines [36];	[28,36,41,42,44]
**47**	agonodepside B	Antimicrobial activity against BS [30]; growth inhibition against human cancer cell lines [36]	[30,36]
**51**	5-bromoagonodepside B	Antimicrobial activity against BS [30]	[30]
**52**	aspergiside B/unguidepside A/aspergillusidone G	-	[25,30,41,44]
**53**	3-bromounguidepside A	-	[30]
**54**	aspergiside A/decarboxyunguidepside A	Antimicrobial activity against BS [30];antimicrobial activity against SA and MRSA [25]; growth inhibition against human cancer cell lines [36]	[25,30,36,41]
**85**	unguidepside C	Antimicrobial activity against BS, ML and SA [36]; growth inhibition against human cancer cell lines [36]	[36]
**87**	agonodepside C	Antimicrobial activity against BS, ML and SA [36]	[36]
**93**	asperdepside A	-	[44]
**Phthalides**			
9*	(3*S*)-3-ethyl-5,7-dihydroxy-3,6-dimethylphthalide	Antimicrobial in SA, MRSA, CA, CN and MG [42]; antimalarial [42]; HB carcinoma cell line (strong cytotoxicity) [42]; antimicrobial activity against SA, MRSA, and MG [25]	[25,38,40,41,42] also isolated from *Rhytidhysteron* sp. [61]
**34**	asperlide	-	[40,41]
**58**	aspergiside C	-	[25,41]
**71**	asperunguislide A	-	[41]
**72**	asperunguislide B	-	[41]
	**Cyclopeptides**		
**16**	unguisin A	-	[28,55,62]
**17**	unguisin B	-	[55]
**18**	unguisin C	-	[63]
**19**	unguisin D	-	[63]
**20**	unguisin E	-	[55] also isolated from *Mucor irregularis and Aspergillus candidus* [64,65]
**Nitrogen-containing Compounds**			
**90**	variotin B	Anti-inflamatory activity [62]	[62]
**91**	coniosulfide E	-	[62]
**Indanones**			
**39**	asperunguisone A	-	[31,41]
**40**	asperunguisone B	-	[31,41]
**Diarylethers**			
**25**	aspergillusether A	HuCCA-1, HepG2, A549, MOLT-3 cancer cell line (weak cytotoxicity) [29]	[29,34]
**41**	aspergillusether B	-	[31]
**42**	aspergillusether C	-	[31,41]
**43**	aspergillusether D	Antimicrobial activity against CA, CN and PM [31]	[31,35,41]
**55**	unguinolic acid	-	[30]
**56**	decarboxyunguinolic acid	-	[30]
**57**	5-chlorounguinolic acid		[30]
**67**	aspergillusether E	Antimicrobial activity against SA, MRSA, CA, CN and MG [38]; cytotoxic activity against Vero cells [38]	[41]
**68**	aspergillusether F	Antimicrobial activity against BC [35]; anti-phytopathogenic activity against CA [35]; inhibition effect on LPS-induced NF-kβ activation [46]. Antimicrobial activity against MRSA, MV, MJ and VP [46]	[35,41,46]
**79**	aspergillusether G	-	[35]
**80**	aspergillusether H	Antimicrobial activity against MT, BC and SA [35];	[35]
**81**	aspergillusether I	Antimicrobial activity against BC and SA [35]; Anti-phytopathogenic activity against CA [35];	[35]
**82**	aspergillusether J	Antimicrobial activity against MT, BC and SA [35]; Anti-phytopathogenic activity against CA [35]; Inhibition effect on LPS-induced NF-kβ activation [46]. Antimicrobial activity against MRSA, MV, MJ and VP [46];	[35,46]
**Pyrone**			
**24**	3-methyl-4-hydroxy-6-(1-trans-methyl-1-propenyl)-2-pyrone	Antimicrobial activity against MT [35];	[29,35,41]
**Orcinol/Orsenillate Derivatives/Phloroglucinol**			
**29**	aspergillusphenol A	Antimicrobial activity against SA and MRSA [31]	[29,31,40,41,44]
**30**	aspergillusphenol B	-	[42]
**36**	methyl orsellinate	-	[31,40,41]
**37**	pilobolusate	Antimicrobial in SA, MRSA, CA, CN and MG [42]; antimalarial [42]; KB cell line (cytotoxicity) [31]	[31,40,41] also isolated from *Pilobolus heterosporus* [66]
**38**	(+)-montagnetol	-	[31,40,41] also isolated from *Roccella montagnei* [67]
**44**	orcinol	-	[31,41]
**66**	aspergillusphenol C	-	[41]
**73**	grifolin A	-	[68]
**74**	grifolin B	DPPH radical scavenging activity [68]	[68]
**89**	1-(2,6-dihydroxy-4-methoxy-3,5-dimethylphenyl)-methylbutan-1-one	Inhibition effect on LPS-induced NF-kβ activation [46]; antimicrobial activity against MV, MJ and VP [46]	[46]
**92**	aspergillusphenol A carboxylic acid	-	[44]
**Benzoic Acid Derivatives**			
**31**	3,5-dibromo-2,4-dihydroxy-6-methyl-benzoic acid methyl ester	-	[42]
**Anthraquinones**			
75*	averantin	-	[68] also isolated from *Aspergillus versicolor* [69]
**76**	7-chloroaveratin	-	[68]
**77**	1′-O-methylaveration	-	[68]
**Chromone**			
**78**	7-hydroxy-2-(2-hydroxypropyl-5-pentyl-chromone	-	[68]
**Sesterterpenoids**			
**59**	asperunguisin A	Cytotoxicity against A549 and HepG2 human cancer cells [26]	[26]
**60**	asperunguisin B	Cytotoxicity against SMMC-7721 human cancer cells [26]	[26]
**61**	asperunguisin C	Cytotoxicity against HT-29, A549, U251, U87, SMMC-7721 and HepG2 human cancer cells [26]	[26]
**62**	asperunguisin D	-	[26]
**63**	asperunguisin E	-	[26]
**64**	asperunguisin F	-	[26]
**65**	aspergilloxide	-	[26]
**Sterols**			
**94**	aspersterol A	Cytotoxicity against cancer cell lines [32]	[32]
**95**	aspersterol B	Anti-inflammatory activity [32]	[32]
**96**	aspersterol C	Anti-inflammatory activity [32]	[32]
**97**	aspersterol D	Anti-inflammatory activity [32]	[32]

## Data Availability

Not applicable.
